# Study of workshop network stability based on pinning control in disturbance environment

**DOI:** 10.1038/s41598-023-32562-z

**Published:** 2023-04-03

**Authors:** Xiaojuan Li, Gaojian Cui, Shunmin Li, Fangyuan Zhang

**Affiliations:** 1 Xinjiang University School of Mechanical Engineering, Xinjiang, Urumqi, 830000 China; 2 Xinjiang University School of Business, Xinjiang, Urumqi, 830000 China; 3 Xinjiang Research Institute of Industrial Economy and Information Technology, Postdoctoral Research Station, Xinjiang, Urumqi, 830000 China

**Keywords:** Mechanical engineering, Mathematics and computing

## Abstract

In the production process, the manufacturing behavior and all the essential factors are affected by several disturbance factors, showing a complex dynamic fluctuation law. It makes the stability control process a difficult problem in environmental constraints. In this paper, the workshop production process is considered, and an improved coupled map lattice workshop production network state model is proposed. On this basis, the controller with the function of resource load protection is designed, and the network state model of the workshop based on the pinning control is developed. Three kinds of stability control strategies, SAC (Self-adaption Control) , SC (Self-acting Control) and PC (Pinning Control) , are designed based on disturbance triggering behavior and node state transition rules. In addition, two control effect evaluation indexes, RTS (Recovery Time Steps) and NFT (Node Failure Times) are designed. Considering the actual production data of diesel fuel injection system parts production workshop as example, the model is simulated and verified. The results show that under different disturbance intensities, compared with the SAC strategy, the RTS-Average value of the PC strategy is reduced by 29.83% on average, and the NFT-Average values are reduced by 46.9% on average. This proves that the pinning control strategy has certain advantages in controlling time length and propagation scale of disturbance propagation.

## Introduction

Stability control of workshop production process is one of the most important management objectives of every discrete manufacturing enterprise. In the "Internet Plus" era, enterprises have begun to customize their products to customers, which results in a low return rate of order procurement, large number of products in process and frequent disturbance in production process. In the production process, the manufacturing behavior and the whole factors of human, machine, material, method and environment are affected by the dynamic changes of several uncertain factors, showing a complex fluctuation law. The stability control of the production process is facing great challenges, which has become one of the key issues concerned by researchers.

In the research of manufacturing system stability, Yang et al.24^1^ developed a brittle risk entropy function for disturbance factors in mixed-line production in order to characterize the system stability. They established a system optimization model in combination with the processing cost factors to improve the system stability. Li et al.^2^ proposed a decision method based on the workpiece processing requirements and the production network stability index to evaluate the workpiece processing characteristics. Xiang et al.^2^ proposed a decision method based on the workpiece processing requirements and the stability index of the production network to solve the problem of instability of the production network caused by the addition of new workpiece. Zou et al.^3^ proposed the concept of virtual ideal clean system for production systems with complex structures, and established an event-driven transient analysis model. Li^4^ proposed an event driven bottleneck identification method, and verify the efficiency of the method by developing a Markov chain. In view of the difficulty in quantitative evaluation of manufacturing equipment brittleness, Liu et al.^5^ proposed a quantitative evaluation method of equipment brittleness, which is based on the combination of performance parameters characterizing equipment status and the principle of brittleness risk entropy. Yin et al.^6^ proposed a method for modeling and performance analysis of production flow network of discrete manufacturing workshops based on complex network theory, aiming at the problem that the production environment of discrete manufacturing workshops is complex, the processing tasks are changeable, and the production anomalies are frequent, which makes it difficult for workshop production logistics to support the optimal operation of production lines and efficient execution of tasks. In order to scientifically evaluate and timely respond to the impact of the disturbance events on the system performance, Wang et al.^7^ developed an event driven pipeline modeling and quality inspection machine configuration optimization. Ti et al.^8^ designed a mixed integer linear programming model and an iterative solution method based on predictive responsive rescheduling strategy to solve the rescheduling problem of flow shop with mixed blocking constraints. In view of the uncertain factors such as machine faults existing in the production process of the workshop, Fu et al.^9^ adopted the binary tree method to construct the stability scheduling optimization model of the workshop under the condition of machine faults which reduces the influence of rescheduling on the production order. Scholars have carried out a lot of research on the theoretical models and solutions of complex production processes, which have provided references for the research work of this paper. However, most of the current studies mainly focus on discussing the fluctuation characteristics and change rules of production parameters under the influence of process correlation when some production attributes are subject to external disturbance or self-change under the disturbance environment. There is still a lack of in-depth research on the polymorphism and evolution process of workshop production resource nodes under the disturbance environment, as well as the relationship between production process stability control and production system topology characteristics.

For the study on the method of pinning control in complex networks, Sun et al.^10^ proposed a pinning control scheme by studying the fixed synchronization scheme of random multi-layer networks under intra-layer coupling disturbance and random noise which saved the control cost. Li et al.^11^ considered the connection of cooperation and competition between neuron nodes in the coupled neural network model, designed a fixed discontinuous controller that made full use of the mode information which obtain an effective criterion to ensure the stability of binary synchronization error state and realize that all network nodes could bidirectional synchronization of the target node state. Liu et al.^12^ efficiently identified the key nodes and node groups in the network by taking advantage of the feature that the node with the largest minimum eigenvalue of Laplacian deleted matrix is more important. They deduced that the optimal control node group is not completely composed of nodes with the highest importance. Hu et al.^13^ studied the quasi-synchronization problem of uncertain complex networks based on event-triggered communication strategies in time-varying topology, and proposed a periodic time-varying network topology applicable to CDNS (Uncertain Complex Dynamical Networks) . Wang et al.^14^ solved the synchronization problem of nonlinear coupled complex networks through event-dependent aperiodic intermittent fixed control, and established an event-dependent intermittent control strategy based on region division.

With the development of complex network theory, the control of complex systems based on this theory has become a new research hotspot. Pinning control is to control some nodes in the network and take the difference of the output of these nodes as the controller so as to achieve the effect of global control. Based on this idea, Fang et al.^15^ used different containment and control strategies to explore the dynamic change rules of the brain network model. They provided a new thinking perspective for the treatment of brain diseases combined with the analysis results. Zhang^16^ achieved the expected traffic flow control of urban sections close to the stable situation by combining the signal lamp time of key intersections with the containment controller. Wang et al.^17^ applied the linear feedback control and containment control theory in order to study the supply chain network with multi-delay and delay coupling. In addition, they performed network synchronization by controlling one node. Jiang ^18^ evaluated the effect of synchronous speed of all the generators by using the pinning control strategy to carry out pin adaptive control on complex power grid. Liu et al.^19^ proposed a novel pinning control strategy for mixed platoon to achieve indirect control of manual driving vehicles. Yao^20^ proposed the brain network containment control model by studying the specific strategies that drive the brain network to change from one state to another. Based on the information physical description of mixed traffic, Sun et al.^21^ proposed a CPS(Cyber-Physical Systems)-based mixed traffic containment control method under dynamic physical topology which can indirectly control the traditional people driving in the traffic to optimize the entire traffic system. In summary, the containment control method has been applied in brain network, transportation, supply chain, electricity and other fields. However, there are few reports on its application to the stability control of manufacturing workshop.

To sum up, this paper adopts the method of containment control to study the stability control of the production line, which has better advantages. This paper takes the production process of the workshop as the research object, proposes an improved coupled map lattice workshop production network state model, which is triggered by abnormal manufacturing behavior and driven by production data, designs a controller with resource load protection, and constructs a network state model of the workshop based on containment control, which can more accurately analyze the impact of disturbance. Three stability control strategies (SAC, SC, PC) and two evaluation indicators representing the control effect of spatiotemporal characteristics are designed based on the disturbance triggering behavior and node state transition rules, so that the stability performance analysis and control of the production line under the influence of disturbance can be evaluated more quickly. The simulation and validation analysis of the constructed containment control model provides a theoretical basis for improving the efficiency of the recovery stability of the workshop network under the disturbance environment.

## Development of a network model of pinning control in the workshop

In the production process of the workshop, there are many uncertain factors from the external environment, which can interfere with the stable execution of the production plan in the workshop. For instance, disturbances such as emergency order insertion, change in delivery date, change in order demand and other excitation, will lead to the increase of articles being processed in the production process, completion time delay and other conditions that will have a serious direct or indirect impact on the stability of the production process. The pinning control method consists in directly exerting the control effect on a small number of nodes in the network, and transferring the control effect to the whole complex network through the coupling effect between nodes, so as to realize the effect of indirectly controlling the whole complex network. Therefore, based on the thought and theory of the pinning control, the diversion control state model and stability control strategy of the workshop are developed. Based on the state model of the production network of the workshop, the state model of the pinning control of the workshop is constructed, and the controller with the function of resource load protection is designed, so that the workshop production network can reach the desired stable state within a certain period of time.

### Workshop production network state model

The state of workshop production network is composed of the state of manufacturing resource node, which is the basic unit of the network. Therefore, the state of resource node is defined. In the actual production process, when the whole production network is in a stable state, it is clear that the material flow between the manufacturing resources is in a stable and smooth state. That is, the materials of manufacturing equipment are not blocked. By defining the node material smoothness, the manufacturing resource node status is represented. The node material characterizes the manufacturing resource node state by the smoothness of the node. The node material smoothness is defined as the ratio of the average arrival rate of materials at the manufacturing resource node to the rate of workpiece processing at this node in the production process:

1 $$\mathrm{where}: {\mathcalligra{x}}_{\mathcalligra{i}}\left(\mathcalligra{t}\right)=\frac{{\sum }_{j}^{N}{v}_{ij}}{{\sum }_{j}^{N}\overline{{v}_{ij}}}$$

${\mathcalligra{x}}_{\mathcalligra{i}}\left(\mathcalligra{t}\right)$: material smoothness of resource node $\mathcalligra{i}$ at time $\mathcalligra{t}$; ${\mathcal{V}}_{\mathcalligra{i}\mathcalligra{j}}$: processing rate of the $\mathcalligra{j}$ th workpiece processed by resource node $\mathcalligra{i}$; $\overline{{\mathcal{V}}_{ij}}$: the average rate at which the $\mathcalligra{j}$ th material reaches the resource node $\mathcalligra{i}$; $\mathcal{N}$: a workpiece processed in an inherent resource node $\mathcalligra{i}$ in a unit time.

When the average arrival rate of node materials is greater than the rate of workpiece processing of a node, the resource node is in the overload processing state, and the node is in the failure state. When the average arrival rate of node materials is less than the rate of workpiece processing of a node, the resource node is in the under-load state, and the node is in the stable state. When the average arrival rate of node materials is equal to the rate of workpiece processing of node, the node is in an ideal processing state, where the utilization rate of resources and equipment is the highest, and the node is also in a stable state.

### Description of the pinning control network model

The production process of complex products has complex kinetic characteristics. The coupled map lattice model can fully characterize the complex dynamics of the system^23^. This paper uses the model to describe the dynamic change process of resource nodes in the workshop production network. A workshop production network state model is established as:

2 $${\mathcalligra{x}}_{\mathcalligra{i}}\left(\mathcalligra{t}+1\right)=\left|(1-\varepsilon )f({x}_{i}(t))+\varepsilon {\sum }_{j=1,j\ne i}^{n}{a}_{ji}{w}_{ji}\frac{f({x}_{j}(t))}{{s}_{i}^{in}}\right|+R$$

${\mathcalligra{x}}_{\mathcalligra{i}}\left(\mathcalligra{t}+1\right)$: when the manufacturing resource node $\mathcalligra{i}$ is in the state of the time of $\mathcalligra{t}+1$, the state is composed of the dynamic evolution state of the node itself and the coupling effect between nodes in the network. When the disturbance action is used for the node, the influence of the disturbance on the node state should be increased; $f:\mathcal{R}\times {\mathcal{R}}^{\mathcalligra{n}}\to {\mathcal{R}}^{\mathcalligra{n}}$: The workshop contains a large number of resource node, not only does it exhibit a constant periodic motion with complex interactions and dependencies between nodes, but also a form of universal significance, i.e., disordered chaos. The nonlinear function f (·) can represent a single workstation in the network representing a chaotic dynamical system. Selecting a chaotic mapping logistic model:$f\left(\mathcalligra{x}\right)=4\mathcalligra{x}\left(1-\mathcalligra{x}\right)$, whose physical meaning can be characterized as the evolutionary law of the workstation node productivity constraint, $f\left(\mathcalligra{x}\right)=4\mathcalligra{x}\left(1-\mathcalligra{x}\right)$, $0<\mathcalligra{x}<1$, $0<f\left(\mathcalligra{x}\right)<1$; $\varepsilon \in \left(\mathrm{0,1}\right)$: the task inflow coupling coefficient which represents the coupling effect of the upstream node state on the downstream node state; $\mathcalligra{n}$: the total number of manufacturing resource nodes included in the production network; $A={\left({\mathcalligra{a}}_{\mathcalligra{j}\mathcalligra{i}}\right)}_{\mathcalligra{n}\times \mathcalligra{n}}$: the adjacency matrix of this production network model. If the finished workpiece in manufacturing equipment nodes $\mathcalligra{j}$ flows to the nodes $\mathcalligra{i}\left(\mathcalligra{i}\ne \mathcalligra{j}\right)$ for processing, then ${\mathcalligra{a}}_{\mathcalligra{j}\mathcalligra{i}}\ne 1$, otherwise, ${\mathcalligra{a}}_{\mathcalligra{j}\mathcalligra{i}}=0$, which considers that the network model is a directed network, and thus ${\mathcalligra{a}}_{\mathcalligra{i}\mathcalligra{j}}\ne {\mathcalligra{a}}_{\mathcalligra{j}\mathcalligra{i}}$. Combined with the production characteristics of the workshop, there is no connection between the node and itself; ${\mathcal{W}}_{\mathcalligra{j}\mathcalligra{i}}$: the weight of the edge. It indicates the workpiece to be processed from the node to the node, which occupies the processing time of the node; ${S}_{\mathcalligra{i}}^{\mathcalligra{i}\mathcalligra{n}}$: the input strength of manufacturing resource node $\mathcalligra{i}$, representing the sum of edge weights of the resource node $\mathcalligra{i}$; R: the influence of the disturbance factors on the node state.

### Description of the pinning control network model

The state change process of the node in the network is first defined as:

3 $${\mathcalligra{x}}_{\mathcalligra{i}}=f({x}_{i}(t))-c{\sum }_{j=1}^{N}{L}_{ij}h({x}_{j}(t))$$

where ${\mathcalligra{x}}_{\mathcalligra{i}}$ is the state of node $\mathcalligra{i}$, $f$ is the dynamic model of the node itself, ${\mathcal{L}}_{\mathcalligra{i}\mathcalligra{j}}$ represents the connection relationship between nodes in the network so as to determine the coupling effect between nodes, $\mathcalligra{h}\left({\mathcalligra{x}}_{\mathcalligra{j}}\right)$ is the output function, and $\mathcalligra{c}$ is the coupling strength between nodes.

When the states of all the nodes in the network evolve to the form expressed in Eq. (4), the network achieves stability.

4 $$\underset{t\to \infty }{lim}({x}_{i}(t)-{x}_{j}(t))=0,i,j=\mathrm{1,2},...,N$$

The pining control model for complex network is defined by:

5 $${\dot{x}}_{i}=f({x}_{i}(t))-c{\sum }_{j=1}^{N}{L}_{ij}h({x}_{j}(t))+{u}_{i},i=\mathrm{1,2},...,N$$

In Eq. (5), the controller is defined as:

6 $${u}_{i}={\delta }_{i}({g}_{i}({x}_{i})-{g}_{i}(s)),i=\mathrm{1,2},...,N$$

where ${\mathcalligra{g}}_{\mathcalligra{i}}$ is the input pinning function of the control node, ${\delta }_{\mathcalligra{i}}$ is the judgment coefficient of whether the node is pinned down and controlled (a value of 1 means the node is under control, and a value of 0 means the node is not under control), and $s\left(\mathcalligra{t}\right)$ is the network trajectory of mathematical expectation, expressed as:

7 $$\dot{s}=f(s(t))$$

In the pinning control steady state, all the nodes in the network are stabilized to the expectation state by pinning control:

8 $$\underset{t\to \infty }{lim}({x}_{i}(t)-s(t))=0,i,j=\mathrm{1,2},...,N$$

### Workshop pinning control network state model

Rank the importance of the resource nodes of the production network of the workshop, select the top $l$ nodes and set the controller, ${i}_{1},{i}_{2},\dots ,{i}_{l}$ is set as the selected node to apply the controller. $\mathrm{f}(0<f<1)$ is the proportion of the number of pinned nodes in the total number of nodes in the network.

Based on the state model of the workshop production network and the pinning control network model, the state model of the pinning control network of the workshop production network with load protection regulation is established.

9 $$\left\{\begin{array}{c}{x}_{{i}_{k}}(t+1)=\left|(1-\varepsilon )f({x}_{{i}_{k}}(t))+\varepsilon {\sum }_{j=1,j\ne i}^{N}{a}_{j{i}_{k}}{w}_{j{i}_{k}}\frac{f({x}_{j}(t))}{{s}_{i}^{in}}\right|+{u}_{{i}_{k}},k=\mathrm{1,2},...,l\\ {x}_{{i}_{k}}(t+1)=\left|(1-\varepsilon )f({x}_{{i}_{k}}(t))+\varepsilon {\sum }_{j=1,j\ne i}^{N}{a}_{j{i}_{k}}{w}_{j{i}_{k}}\frac{f({x}_{j}(t))}{{s}_{i}^{in}}\right|,k=l+1,l+2,...,N\end{array}\right.$$

where ${\mathcal{U}}_{{i}_{k}}$ is the controller set for the pinned node.

The pinning controller in the production network state model of the workshop is designed as negative feedback control. The deviation between the actual state and the expected stable state of the node at this time is used as feedback to set the controller.

### Design of the controller

The controller is designed as follows:

10 $${u}_{{i}_{k}}=\left\{\begin{array}{l}-cd({x}_{{i}_{k}}(t)-s(t)),1\le k\le l\\ 0,\text{ others}\end{array}\right.$$

11 $$d=\left\{\begin{array}{c}1,{x}_{{i}_{k}}(t)>s(t)\\ 0,{x}_{{i}_{k}}(t)\le s(t)\end{array}\right.$$

where $\mathcalligra{c}$ is the control strength coefficient and $\mathcalligra{d}$ is the control effect judgment coefficient.

In this controller, the deviation between the actual state and the expected stable state of the node in the disturbed environment is used as the feedback of the controller. Combined with the actual characteristics of the production process, when the manufacturing node state is in a stable state but the node efficiency is not high, that is when ${\mathcalligra{x}}_{{\mathcalligra{i}}_{\mathcalligra{k}}}\left(\mathcalligra{t}\right)\le s\left(\mathcalligra{t}\right)$ , the control state of the node tends to the desired state of the node, resulting in the increase of the control state of the node to the downstream node, through the state coupling between nodes, which is very likely to aggravate the deterioration of the state of the disturbed node. Therefore, a judgment coefficient is set in the controller. In addition, by judging the state of the controlled node and then deciding whether to control it, this method is more in line with the actual characteristics of the workshop production process.

### Determination of the network state stability

**Figure 1:**
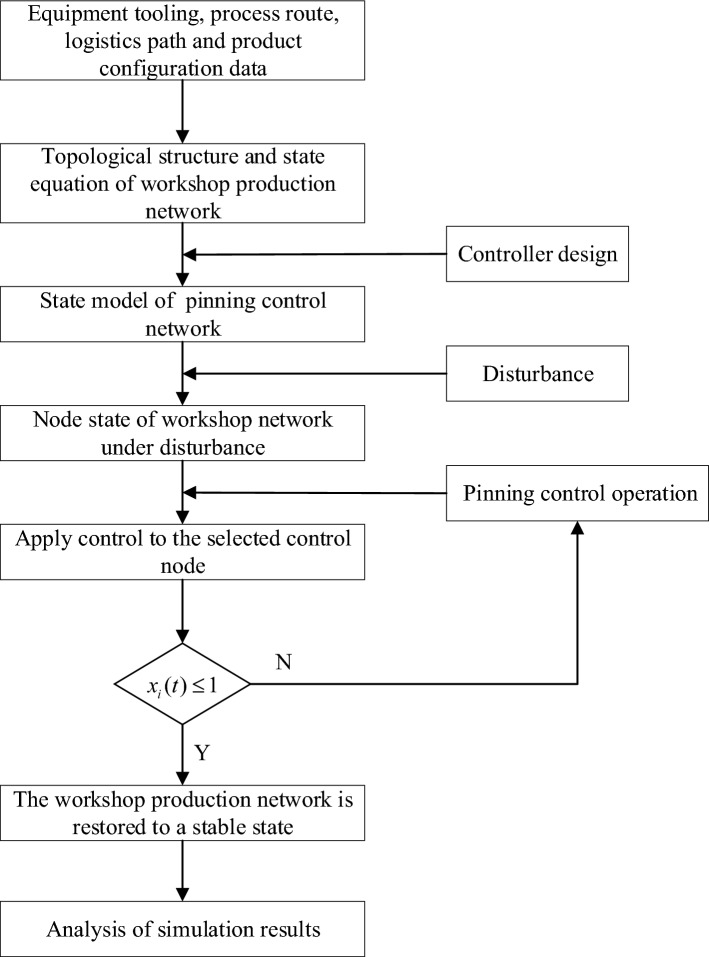
Flowchart of the recovery stability of the pinning control network.

The resource node status of the workshop production network is the material smoothness of the node. By analyzing the actual situation of the production process, it shows that when the production network is in a stable state, the state of each resources nodes is in a stable state. In other words, the resources nodes state is in the (0,1) interval. Simultaneously, the network state model established in combination with the characteristics of production network has also its own characteristics. When the network state recovers from disturbance to stable state, the network will maintain its stable state operation without new disturbance action. Therefore, considering the actual situation when the production process of the actual workshop is stable, combined with the characteristics of the established model, the stability of the pinning control network is defined as follows: when the state value of the node is ${\mathcalligra{x}}_{\mathcalligra{i}}\left(\mathcalligra{t}\right)>1$ , the state of the node is in an overload state, and the state of the node is unstable; when the node is in an unstable state, the state value of the node is ${\mathcalligra{x}}_{\mathcalligra{i}}\left(\mathcalligra{t}\right)\le 1$ which is under control, and the node then returns to a stable state. When all the nodes state value in the network are ${\mathcalligra{x}}_{\mathcalligra{i}}\left(\mathcalligra{t}\right)\le 1$ , the overall state of the network also returns to a stable state. The network restoration stability process is shown in Fig. 1.

## Stability control strategy and evaluation index

### Control strategy design

Aiming at the recovery process of workshop network stability, three different control strategies are proposed: the Self-adaption Control (SAC), Self-acting Control (SC) and Pinning Control (PC). This is performed by comparing the control effects of network recovery stability in different strategies, analyzing the effect of the pinning control method on the recovery of workshop network stability, and tapping the potential action law to make the workshop production control more effective.

- (1) SAC: for the disturbance, it depends on the buffer link existing in the production process to perform self-repair, and does not use any control strategy.
- (2) SC: controls the disturbed nodes. When the workshop production process is disturbed, it can timely and accurately locate the disturbance and subsequent propagation chain information.
- (3) PC: it is used to control disturbed nodes and the key nodes of the system. The number of control nodes is 10% of the total number of the network nodes.

Combined with this method, in the Matlab environment, the production network state simulation under disturbance environment is carried out for the three control strategies. In addition, the operation mechanism of workshop production process hidden behind the simulation data results, is analyzed, so as to help obtain more efficient workshop production management and control decisions.

### Stability evaluation index of pinning control network

- (1) Recovery Time Steps for network (RTS)

RTS is defined as the number of units of time between the moment a disturbance occurs in the production network and the moment when the network returns to stability. This index can measure the control effect of control decision on the stability of workshop network under disturbance from the perspective of time length.

- (2) Network node failure times (NFT)

NFT is defined as the sum of the number of network failure nodes counted per unit time in the production network, from the moment of disturbance to the moment when the network restores stability. This index can characterize the influence intensity of disturbance from the perspective of space, and measure the efficiency of the control from the perspective of the scale of disturbance diffusion.

## Case analysis

### Basic data

Considering the production process of diesel engine fuel injection system workshop of a subsidiary company of southwest Shipping Group as the object of the study, the following raw data are from the literature24^24^. The machining workshop has 20 kinds of manufacturing resources in total, mainly for 9 types of workpiece processing. In contrast to the regular scheduling example, the numbers of processing and process flow of each type of workpiece in the workshop are different. A comparison between the workshop production equipment numbers is shown in Table 1. The processing technology of various workpieces and the number of workpieces are shown in Table 2. The characteristic parameters of the workshop network are presented in Table 3.

**Table 1:** Comparison of workshop production equipment numbers.

I	II	III	IV	V	VI	VII	VIII	IX	X
Crossed platform	Semi-finishing lathe	Cleaning trough	Overspeed machine	Flaw detector	Rough turning lathe	Dynamic balancing machine	Inspection bench	Finish turning lathe	Fine milling milling machine

XI	XII	XIII	IX	XV	XVI	XVII	XVIII	XIX	XX
Bench	Deoxidization units	Laser cutting plotter	Heat treatment furnace	Numerical control lathe	CNC milling machine	Boring lathe	Milling machine	Paint spraying equipment	Drilling machine

**Table 2:** Workpiece manufacturing data of workshop.

Artifacts	Number of artifacts	Workpiece processing technology (processing time)/min							
$J_{1}$	8	$M_{1}$ (8.5)	$M_{6}$ (18)	$M_{12}$ (12)	$M_{9}$ (24)	$M_{1}$ (6)	$M_{16}$(18.5)	$M_{20}$(18.5)	$M_{10}$(14.5)
		$M_{1}$ (6.5)	$M_{18}$ (1)	$M_{11}$ (20)	$M_{12}$(12.5)				
$J_{2}$	8	$M_{12}$(12.5)	$M_{1}$ (14)	$M_{6}$ (25)	$M_{11}$ (8)	$M_{9}$ (23)	$M_{2}$ (15)	$M_{1}$ (15)	$M_{18}$(32.5)
		$M_{20}$(18.5)	$M_{9}$ (7)	$M_{1}$ (8)	$M_{20}$(20.5)	$M_{18}$(18.5)	$M_{10}$ (5)	$M_{20}$ (27)	$M_{12}$(28.5)
		$M_{11}$ (39)	$M_{18}$ (9)	$M_{11}$ (7)					
$J_{3}$	3	$M_{11}$ (4.5)	$M_{17}$(12.5)	$M_{5}$ (6.5)	$M_{17}$ (5)	$M_{11}$ (2.5)	$M_{17}$ (5.5)	$M_{20}$ (1.5)	$M_{19}$ (2)
		$$M_{11}$$ (3)							
$J_{4}$	5	$M_{1}$ _(9)_	$M_{6}$ _(24)_	$M_{9}$ _(24)_	$M_{1}$ _(9.5)_	$M_{17}$_(16.5)_	$M_{16}$_(27.5)_	$M_{20}$ _(6)_	$M_{11}$ _(7)_
		$M_{3}$ _(9.5)_							
$J_{5}$	3	$M_{1}$ _(3)_	$M_{6}$ _(8)_	$M_{9}$ _(15)_	$M_{1}$ _(2)_	$M_{10}$ _(16)_	$M_{11}$ _(2)_	$M_{12}$ _(3)_	
$J_{6}$	5	$M_{9}$ _(16.5)_	$M_{11}$ _(8)_						
$J_{7}$	2	$M_{20}$ _(1.5)_	$M_{6}$ _(7)_	$M_{14}$ _(1.5)_	$M_{8}$ _(2)_	$M_{15}$ _(8.5)_	$M_{17}$ _(3.5)_	$M_{16}$ _(22)_	$M_{14}$ _(3.5)_
		$M_{15}$ _(10)_	$M_{16}$_(12.5)_	$M_{7}$ _(3)_	$M_{4}$ _(3.5)_	$M_{15}$ _(4.5)_	$M_{16}$_(16.5)_	$M_{15}$_(14.5)_	$M_{16}$ _(5)_
		$M_{11}$ _(3)_	$M_{7}$ _(2.5)_						
$J_{8}$	15	$M_{1}$ _(5)_	$M_{6}$ _(13)_	$M_{9}$_(24.5)_	$M_{16}$_(17.5)_	$M_{20}$ _(5.5)_	$M_{11}$ _(8)_		
$J_{9}$	10	$M_{5}$ _(23)_	$M_{18}$ _(16)_	$M_{1}$ _(4)_	$M_{20}$_(10.5)_	$M_{11}$ _(3)_	$M_{13}$ _(5)_

**Table 3:** Network parameters of production network equipment node in workshop.

Node number	Degree value	In degree	Out of degree	Into the intensity	Out of intensity	Clustering coefficient	Betweenness	The upstream nodes	The downstream nodes
0	9	0	9	0	626	0	0	/	1,5,9,11,12,20
1	24	12	12	686.5	1498.5	0.211	0.1209	0,2,9,10,12,18	6,10,16,17,18,20
2	2	1	1	120	120	0.5	0	9	1
3	1	1	0	47.5	0	0	0	11	–
4	2	1	1	7	9	0	0.0491	7	15
5	4	2	2	249.5	175	0	0.0055	0,17	17,18
6	12	6	6	697	695.5	0.4	0.0325	1,20	9,11,12,14
7	3	2	1	11	7	0.166	0.0465	11,16	4
8	2	1	1	4	17	0.5	0	14	15
9	14	7	7	1047	588	0.321	0.0796	0,6,11,12,20	1,2,11,16
10	6	3	3	204	274	0.666	0.0061	1,18,20	1,11,20
11	21	13	8	859	421.5	0.128	0.2627	0,6,9,10,12,16,17,18,19,20	3,7,9,12,13,17,18
12	7	4	3	433	616	0.466	0.0103	0,6,11,20	1,9,11
13	1	1	0	50	0	0	0	11	–
14	5	2	3	10	172	0.25	0.0546	6,16	8,15,20
15	8	4	4	75	75	0.15	0.1526	4,8,14,16	16,17
16	13	7	6	660	160.5	0.25	0.1885	1,9,15,17	7,11,14,15,20
17	10	5	5	158.5	213	0.233	0.1494	1,5,11,15	5,11,16,20
18	10	5	5	648	444	0.35	0.0422	1,5,11,20	1,10,11,20
19	2	1	1	6	9	0.5	0	20	11
20	18	9	9	901	753	0.25	0.181	0,1,10,14,16,17,18	6,9,10,11,12,18,19

### Ranking the importance of nodes

Using the method of carrier (BMW, Best Worst Method) and index weight determining method (CRITIC, Criteria Importance Though Intercrieria Correlation)^25^ the importance of nodes in a network are ranked. Through the analysis of production network data, the order of importance of nodes in the network is calculated (cf. Table 4). The top ten important nodes are 1–20–11–9–6–16–7–8–9–10 in sequence. Node 9 has deviation from the enterprise conclusion, which is referred to as the hidden key node mined through network model association relationship.

**Table 4:** Ranking of node importance in workshop network.

Ranking	Node	Criticality/%
1	1	0.884
2	20	0.699
3	11	0.673
4	9	0.656
5	6	0.548
6	16	0.459
7	18	0.431
8	12	0.427
9	17	0.225
10	10	0.208
11	5	0.173
12	15	0.139
13	2	0.094
14	14	0.057
15	7	0.041
16	8	0.030
17	4	0.0284
18	19	0.028
19	13	0.0279
20	3	0.0271

### Stability analysis of workshop network under three control strategies

By applying a disturbance with a strength R of rank 1 to 6 to the nodes in the network, the control effects of restoring the stability of the network from the disturbance state under different control strategies, are compared. The Parameters in node state calculation are ε = 0.5 and *c* = 0.2. Matlab is used to conduct the numerical simulation of node state of production network. Under the condition of only changing the disturbance intensity R, 2640 times of simulation are conducted, while each simulation consists of the whole process from network disturbance to stability recovery. According to the simulation, the node status data of the production network from disturbed to restored, are obtained. By counting the RTS and NFT indexes of the network from disturbance to recovery, the control effects of the whole network from disturbance to recovery are compared under different control strategies. Through the analysis of the obtained data, the disturbance propagation and control mechanism hidden behind the data are explored.

- 1. Average performance analysis of network recovery stability under different control strategies.

In the experiment, all the nodes in the network are subjected to the same R value, and the disturbed nodes are controlled to restore the network from the disturbed state to the stable state. The average RTS index and average NFT index of all the nodes are obtained, namely the average network performance of a control strategy under the disturbance condition. The RTS-Average and NFT-Average values of network stability under different control strategies are shown in Tables 5 and 6, respectively.

**Table 5:** Comparison of RTS-Average of network stability restoration under different control strategies.

Strategies	Strength (R)					
	**1**	**2**	**3**	**4**	**5**	**6**
SAC strategies	3.25	3.85	4.7	5.75	7.3	8.55
SC strategies	1.65	3.4	3.85	4.4	5.3	5.7
PC strategies control node 1	1.65	3.4	3.85	4.25	5.05	5.4
PC strategies control node 20	1.65	3.4	3.85	4.35	5.15	5.45
PC strategies control node 9	1.65	3.35	3.75	4.15	5.1	5.3
PC strategies control node 5	1.65	3.4	3.85	4.4	5.3	5.7
PC strategies control node 3	1.65	3.4	3.85	4.4	5.3	5.7

**Table 6:** Comparison of the NFT-Average of network stability restoration under different control strategies.

Strategies	Strength (R)					
	1	2	3	4	5	6
SAC strategies	3.25	5.9	10	17	41.4	56.6
SC strategies	1	4.35	7.15	10.9	23.2	29.95
PC strategies control node 1	0.95	4.25	7.3	10	19.45	24.5
PC strategies control node 20	1	4.35	7.05	10.15	19.85	24.1
PC strategies control node 9	1	4.25	6.95	9.95	19.4	24
PC strategies control node 5	1	4.35	7.15	10.9	23.2	29.95
PC strategies control node 3	1	4.3	7.1	10.85	23	29.8

By comparing the network RTS-Average values with different R values (cf. Table 5), it can be seen that the RTS-Average values under SC and PC strategies are significantly reduced, compared with the SAC strategies. Compared with the SAC strategy, the RTS-Average value of the PC strategy decreases by 49.2%, 13%, 20.2%, 27.8%, 30.8% and 38%, respectively. In addition, it can be seen from Table 5 that the RTS-Average value of the network at control node 9 under the PC strategy is better, which is consistent with the conclusion that the node is a hidden key control node in the actual operation of the workshop. An effective mining of such nodes is more conducive to the recovery of the network stability of the workshop.

By comparing the NFT-Average values of networks with different R values (cf. Table 6), it can be seen that the NFT-Average values of the SC and PC strategies are significantly reduced compared with the SAC strategies, and the gap gradually increases with the increase of R values, which is consistent with the trend of RTS-Average values. Compared with the SAC strategy, the NFT-Average value of PC strategy decreases by 70.8%, 27.9%, 30.5%, 41.5%, 53.1% and 57.6%, respectively. The NFT-Average value of the network, from the perspective of the propagation range of disturbance in the network, more clearly shows that the PC strategy with different R values can efficiently suppress the propagation of disturbance in the network, and significantly reduce the propagation range of disturbance.

- 2. The relationship between disturbance intensity R and control intensity coefficient c is analyzed.

When different nodes in the network are separately disturbed, the change relationship between different disturbance intensity R and control intensity coefficient *c* is analyzed, for the same selected restraining control node. Note that 19,200 simulation times are considered for this experiment. Due to the different importance of network node control degree causing different impacts on the stability of the network, in order to eliminate the interference of the factors according to the node important degree of sorting, node 1 and node 3 are selected for the control simulation, and the controlled nodes are compared under different R values when increasing the intensity of the control coefficient *c*, in order to analyze the tendency of the network performance.

1. Analysis of the relationship between R and *c* when pinning control node.

**Figure 2:**
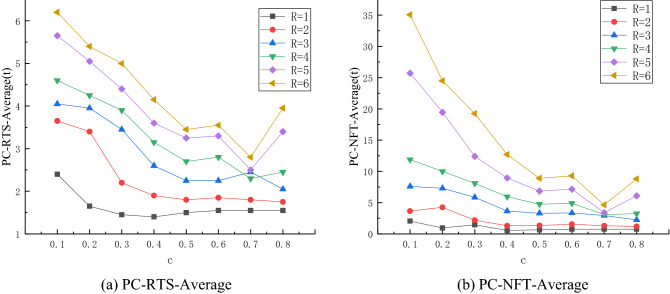
Relationship curve of different R and *c* values when pinning control node 1.

When simulating the containment control node 1, all the nodes in the network are disturbed, and the stable RTS-Average value and NFT-average value of network recovery are obtained. It can be seen from Fig. 2a that, when *c* ∈ [0.1,0.5], the network RTS-Average value corresponding to the same R value presents a downward trend with the increase of *c*, which is consistent with the performance of the actual workshop production process. When *c* ∈ [0.5,0.6], the change of RTS-average value under different R values is relatively smooth, which shows that in this case, increasing the control intensity does not reduce the duration of disturbance in the network under different disturbance intensities. Finally, when *c* ∈ [0.6,0.8], the data fluctuate.

It can be seen that the overall trend in Fig. 2b is consistent with that in Fig. 2a. When *c* ∈ [0.1,0.5], the NFT-average value of the network corresponding to the same R shows a downward trend with the increase of *c*. When *c* ∈ [0.5,0.6], increasing the control intensity does not reduce (or even increases) the propagation scale of disturbance in the network. When *c* ∈ [0.6,0.8], the data fluctuate.

Based on this analysis, the variation relationship between R and *c* of the network when the control node 1 is restrained, can be determined. When* c* ∈ [0.1,0.5], the same R gradually decreases with the increase of *c*. When *c* ∈ [0.5,0.6], the network performance value slightly changes. When *c* ∈ [0.6,0.8], the change trend of the network performance is different for different R values. More precisely, for R = 4,5 and 6, it shows a downward trend at *c* = 0.7 and sharply increases at *c* = 0.8. For R = 3, the change trend of the network performance value is opposite, while the change trend is small when R = 1 and 2. It can be seen that the relationship between R and *c* is complex and nonlinear.

2. Analysis of the relationship between R and c when control node 3 is involved.

**Figure 3:**
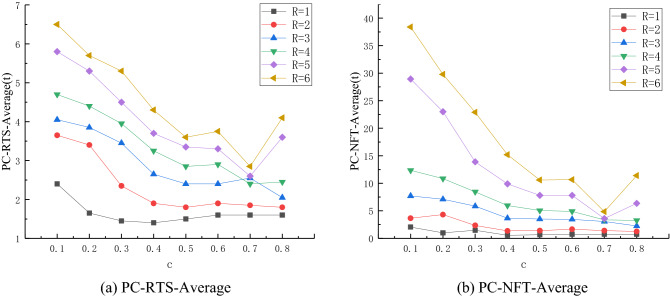
Variation relationship between different R and *c* values at control node 3.

When the simulation contains control node 3, all the nodes in the network are disturbed, and the RTS-Average and NFT-Average values of the restored stability of the network are obtained (cf. Fig. 3). It can be seen from Fig. 3a that, when *c* ∈ [0.1,0.5], the RTS-Average value of the network corresponding to the same R presents a declining trend with the increase of *c*. In addition, the larger R corresponding to the same *c*, the greater the value, which is consistent with the situation of containing node 1 and node 10, and the declining range is [1.4,6.5]. When *c* ∈ [0.5,0.6], the RTS-Average values of networks under different R values slightly change. More precisely, under different disturbance intensities, increasing the control intensity does not reduce the duration of disturbance in the network, and the RTS-Average value change interval is [1.5,3.75]. When *c* = 0.7, the RTS-Average value of the network sharply decreases at R = 4, 5 and 6, and presents an upward trend at R = 3. When R = 1 and 2, the change is flat and slow. When *c* = 0.8, the RTS-Average value of the network sharply decreases at R = 4, 5 and 6, and presents an upward trend at R = 3. The RTS-Average value of the network shows an upward trend at R = 4, 5 and 6, a downward trend at R = 3, and a slight change at R = 1 and 2. The RTS-Average value varies in the range [1.6,4.1]. It can be seen from Fig. 3b that the overall trend is consistent with Fig. 3a. When *c* ∈ [0.1,0.5], the NFT-Average value of the network corresponding to the same R shows a downward trend with the increase of* c*. The larger R corresponding to the same *c* is, the greater the value, and the downward range is [0.55,38.4]. When *c* ∈ [0.5,0.6], the changes of NFT-Average value of the network under different R are relatively smooth, and there is a slight upward trend. That is, under different disturbance intensities, the increase of control intensity does not reduce (or even increases) the propagation scale of disturbance in the network. The variation range of NFT-Average value is [0.65,10.65]. When *c* = 0.7, the NFT-Average value of the network significantly decreases at R = 4, 5 and 6, presents a slight downward trend at R = 3, and slightly changes at R = 1 and 2. When* c* = 0.8, the RTS-Average value of the network shows an upward trend at R = 5 and 6, a downward trend at R = 3, and a slight change at R = 1, 2 and 4. The NFT-Average value varies between 0.8 and 11.4.

- 3. Analysis of the influence of different control intensity coefficient c on the network stability control performance, for the same R.

From the perspective of a single node with different control importance, the influence of different control intensity coefficient c on the network control performance under the same disturbance intensity R, is analyzed. 19,200 simulation times are considered in this experiment. Since R is set to 1 ~ 3, the performance index changes of network recovery and stability show a steady and continuous downward trend when the simulation *c* changes, and therefore the focus is not discussed in this section.

A) R = 4, the performance indicator of network recovery stability changes when *c* changes.

**Figure 4:**
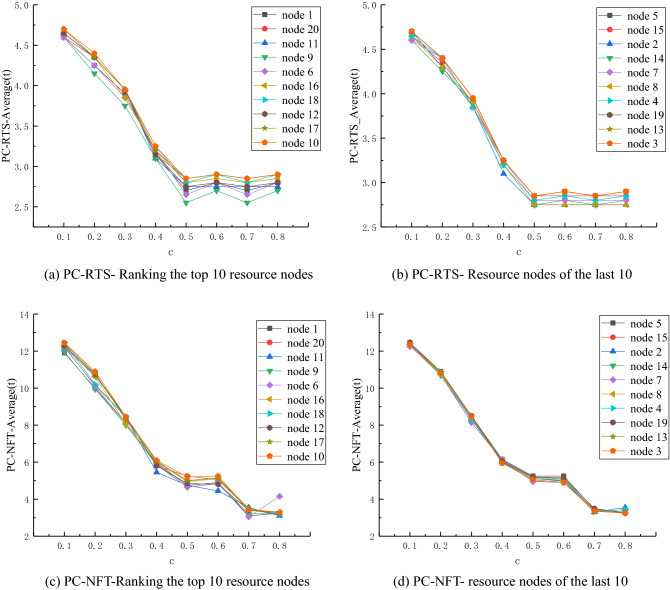
Network performance curve for R = 4 and *c* = 0.1, 0.2, 0.3, 0.4, 0.5, 0.6, 0.7, 0.8.

The coefficient of disturbance intensity is set to R = 4, and *c* = 0.1, 0.2, 0.3, 0.4, 0.5, 0.6, 0.7 and 0.8, in order to study the control network in different nodes. The change of the performance of the network stability returns curve is shown in Fig. 4.

It can be seen from Fig. 4a and Fig. 4b that, when *c* ∈ [0.1,0.5], the RTS-Average value of the network corresponding to different control nodes presents a continuous decline trend with the increase of *c*. The decline interval of Fig. 4a is [2.7,4.7], and that of Fig. 4 (b) is [2.75,4.7]. When *c* ∈ [0.5,0.8], the RTS-average value of the network with different containment control nodes begins to fluctuate.

It can be seen from Fig. 4c and Fig. 4d that, when *c* ∈ [0.1,0.5], the NFT-Average value of the network corresponding to different containment control nodes presents a downward trend with the increase of *c*, wherein (a) and (b) fall within the range of [2.7,4.7] and [2.75,4.7], respectively. When *c* ∈ [0.5,0.6], the NFT-Average value of the network with different containment control nodes slightly increases, where (a) varies between [2.55,2.9], and (b) varies between [2.75,2.9]. When *c* ∈ [0.6,0.8], the NFT-Average value of the network of each node slightly fluctuates.

B) R = 5, the performance indicator of network recovery stability changes when c changes.

Figure 3 shows that the curve of network stability decreases for a turbulence intensity R = 5 and coefficient *c* = 0.1, 0.2, 0.3, 0.4, 0.5, 0.6, 0.7 and 0.8, containing the control network in different nodes.

**Figure 5:**
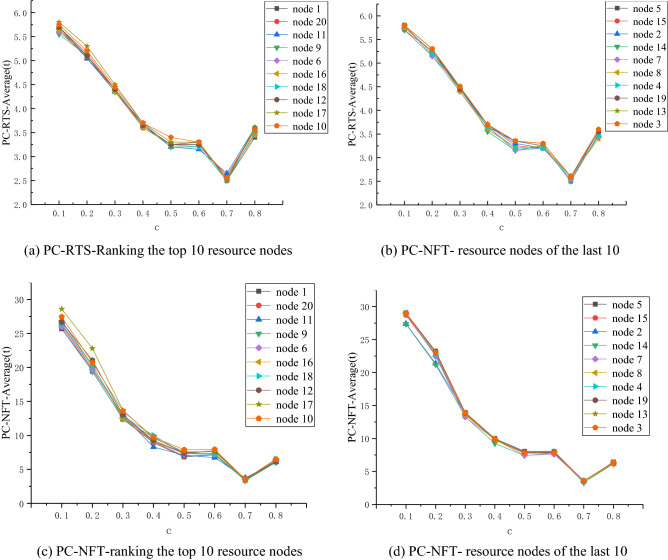
Network performance curve for R = 5 and *c* = 0.1, 0.2, 0.3, 0.4, 0.5, 0.6, 0.7, 0.8.

It can be seen from Fig. 5a and Fig. 5b that, when *c* ∈ [0.1,0.5], the RTS-Average value of the network corresponding to different containment control nodes presents a downward trend with the increase of *c*. When *c* = 0.7, the RTS-Average value of the network of each node slightly decreases. When *c* = 0.8, the RTS-Average value of the network of each node shows an upward trend. Figure 5a shows a change interval of [2.5,3.6], while the variation range of Fig. 5b is [2.5,3.6].

It can be seen from Fig. 5c and Fig. 5d that, when *c* ∈ [0.1,0.5], the NFT-Average value of the network corresponding to different containment control nodes presents a downward trend with the increase of *c*. When *c* ∈ [0.5,0.6], the NFT-Average value of the network with different control nodes slightly increases. The variation range of Fig. 5c is [6.75,7.95], and that of Fig. 5d is [7.4, 8.05]. When* c* = 0.7, the network NFT-Average value of each node has a slight decline. When *c* = 0.8, the network NFT-Average value of each node presents an upward trend. The variation range of Fig. 5c is [3.35,7.95], while that of Fig. 5d is [3.3,8.05].

When R = 5, the overall trend is consistent with that when R = 4. With the increase of *c*, both RTS and NFT of network performance show a decreasing trend. This indicates that there is a threshold value of control intensity coefficient* c* in the network, and it is independent of the disturbance intensity R.

B) R = 6, the performance indicator of network recovery stability changes when c changes.

**Figure 6:**
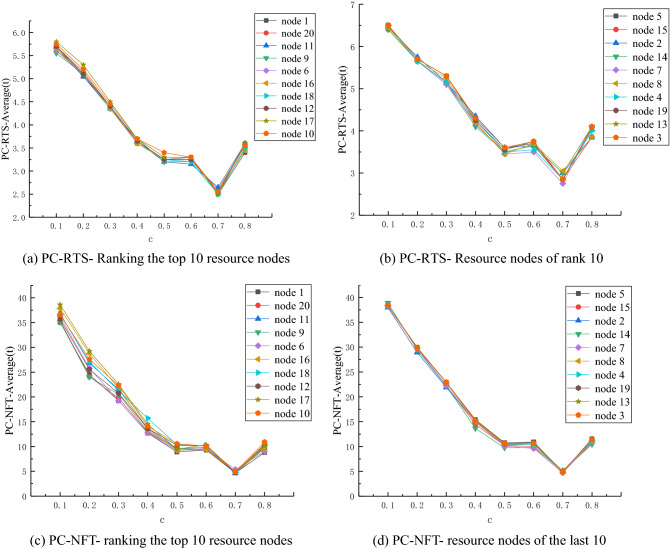
Network performance curve for R = 4 and *c* = 0.1, 0.2, 0.3, 0.4, 0.5, 0.6, 0.7, 0.8.

It can be seen from Fig. 6a and Fig. 6b that, when *c* ∈ [0.1,0.5], the RTS-Average value of networks corresponding to different control nodes presents a decreasing trend with the increase of *c*, with a decreasing range of [3.45,6.45]. When *c* ∈ [0.5,0.6], the RTS-Average value of networks with different control nodes slightly increases, and the variation range is [3.45,3.75]. When *c* = 0.7, the RTS-Average value of the network of each node has a slight decline. When* c* = 0.8, the RTS-Average value of the network of each node presents an upward trend. Among them, the change interval of (a) is [2.8,4.05], and the variation range of (b) is [2.75,4.1].

It can be observed from Fig. 6c and Fig. 6d that, when *c* ∈ [0.1,0.5], the NFT-Average value of the network corresponding to different containment control nodes presents a downward trend with the increase of *c*. When *c* ∈ [0.5,0.6], the NFT-Average value of the network with different control nodes slightly increases. When *c* = 0.7, the network NFT-Average value of each node slightly decreases, and when *c* = 0.8, the network NFT-Average value of each node presents an upward trend.

### Analysis of the simulation results

1. The simulation of the control effects of the three control strategies in a disturbance environment shows that the PC strategy can efficiently control the restoration and stability of the network state. Under different R values, the RTS-Average value of the PC strategy respectively decreases by 49.2%, 13%, 20.2%, 27.8%, 30.8% and 38%, compared with the SAC strategy, while the NFT-Average value is reduced by 70.8%, 27.9%, 30.5%, 41.5%, 53.1% and 57.6%, respectively. The superiority of the PC strategy proposed in this paper is demonstrated in terms of the time length of the perturbation propagation and the propagation scale.
2. Through the discussion and analysis of the control intensity coefficient *c* in the workshop network state model, it can be deduced that, when the control intensity coefficient *c* ∈ [0.1,0.5], the control effect shows a downward trend and gradually recovers to stable. When *c* ∈ [0.5,0.8], the network stability index of each node shows a dynamic fluctuation. When *c* = 0.6, the containment control will slow down the recovery speed of the production network. When *c* = 0.8, the network has an overall performance degradation speed. This is due to the production network stability control function of complicated nonlinear relation between disturbance intensity. Therefore, the proposed method is accurate in identifying the disturbance situation, in order to cooperate with appropriate intensity control function, and therefore it is more conducive to the restoration of workshop production process stability.

## Conclusions

In this paper, an improved coupled image grid workshop production network state model is proposed. The proposed model is triggered by abnormal manufacturing behavior and driven by production data. On this basis, a controller with resource load protection function is designed, and a network state model based on containment control is constructed. The SAC, SC and PC stability control strategies and the RTS and NFT control effect evaluation indexes, are designed combined with disturbance triggering behavior and node state transition rules. The simulation results show that the stability recovery performance of the workshop network under the contained control strategy is better, the RTS-Average value of the PC strategy is reduced by 29.83% on average, and the NFT-Average values are reduced by 46.9% on averages. When the control strength coefficient *c* ∈ [0.5,0.8], the network stability index of each node shows a dynamic fluctuation. In future work, the complex nonlinear relationship between the stability control function of production network and the disturbance intensity will be further explored. This provides a theoretical basis for efficiently carrying out the optimization control of the production process.

## Data Availability

The datasets generated during and/or analyzed during the current study are available from the corresponding author on reasonable request.
